# Supplementary material for: A biodegradable, flexible photonic patch for in vivo phototherapy
Source: Nat Commun. 2023 May 27;14:3069. doi: 10.1038/s41467-023-38554-x (PMC10224912; doi:10.1038/s41467-023-38554-x)
Supplement: Supplementary file 1 — Supplementary information [file 41467_2023_38554_MOESM1_ESM.pdf]

Supporting information

A biodegradable, flexible photonic patch for in vivo phototherapy

Kaicheng Deng[#], Yao Tang[#], Yan Xiao[#], Danni Zhong[#], Hua Zhang, Wen Fang, Liyin Shen, Zhaochuang Wang, Jiazhen Pan, Yuwen Lu, Changming Chen, Yun Gao, Qiao Jin, Lenan Zhuang, Hao Wan, Liujing Zhuang, Ping Wang, Junfeng Zhai, Tanchen Ren, Qiaoling Hu, Meidong Lang, Yue Zhang, Huanan Wang, Min Zhou*, Changyou Gao*, Lei Zhang*, Yang Zhu*

Contents

Supplementary Methods

Supplementary Note 1. Synthesis of CCS@gel.

Supplementary Note 2. Mechanical strength tests.

Supplementary Note 3. Patch substrate degradation in vitro.

Supplementary Note 4. Illumination demonstration on rat liver and hindlimb muscles.

Supplementary Note 5. Demonstration of 445 nm laser transmission.

Supplementary Note 6. Tumor photodynamic therapy in mice.

Supplementary Note 7. Characterization of light field distribution of different optical fibers.

Supplementary Note 8. Intramyocardial injection of Chlorella.

Supplementary Note 9. Immunofluorescent staining.

Supplementary Note 10. RNA-seq analysis.

Supplementary Note 11. ROS accumulation in cardiomyocytes after Chlorella and illumination treatment.

Supplementary Note 12. Phototoxicity assay.

Supplementary Note 13. Proliferation assay of cardiac fibroblasts.

Supplementary Note 14. Electrophysiology and myocardial contractility assay in vitro.

Supplementary Note 15. Measurement of heart rate of iCarP treated dog.

Results

Supplementary Equation 1. Shape of the tapered optical fiber.

Supplementary Figure 1. Fabrication and characterization of PMCL patch.

Supplementary Figure 2. ICarP adhesion and TOF removal.

Supplementary Figure 3. Electric field intensity distribution of light emitting for iCarP with different distance between the TOF tip and air gap tip (*d*).

Supplementary Figure 4. Demonstration of iCarP application on rat hindlimb muscle and liver.

Supplementary Figure 5. ICarP guided light with wavelength of 445 nm onto an ex vivo porcine heart.

Supplementary Figure 6. Effects of tumor photodynamic therapy in mice.

Supplementary Figure 7. Light scattering effects of flat-end optical fibers, tapered optical fibers, and clinically used optical fibers.

Supplementary Figure 8. Evaluation of the potential risks of bleeding and inflammation caused by Chlorella injection.

Supplementary Figure 9. Expression of apoptosis markers in myocardium.

Supplementary Figure 10. RNA-seq analysis of cardiac tissues 1 day after surgery.

Supplementary Figure 11. Effect of Chlorella and light on ROS accumulation in cardiomyocytes, which was stained by DCFH-DA.

Supplementary Figure 12. Effect of extended illumination on cardiomyocyte viability.

Supplementary Figure 13. Effects of Chlorella and light on myocardial electrophysiology and myocardial contractility in vitro.

Supplementary Figure 14. Effect of iCarP on heart rate of a dog.

Supplementary Methods

Supplementary Note 1. Synthesis of CCS@gel.

CCS@gel was synthesized as previously described[1]. Briefly, the catechol-functionalized chitosan (CCS) was synthesized via the 1-ethyl-3-(3-dimethylaminopropyl) carbodiimide catalyzed amidation between the carboxyl group of hydrocaffeic acid and amino groups of chitosan, as the catechol substitution degree of 1% calculated from ^{1}H NMR analysis. The dibenzaldehyde-terminated polyethylene glycol (DB-PEG2000) was prepared by esterification of dihydroxyl-terminated PEG with 4-formylbenzoic acid, and PEG chains were modified with aldehyde groups at both ends. Then, a tissue-adhesive hydrogel (CCS@gel) was constructed in situ by mixing equal volume of CCS and DB-PEG2000 solutions, via Schiff base reactions between the amino groups of CCS and aldehyde groups of DB-PEG2000. During gelation, the catechol moieties of CCS could also react with amino groups on chitosan chains or tissue surface via Michael addition or Schiff base linkages, generating the network and offering tissue adhesiveness.

Supplementary Note 2. Mechanical strength tests.

Crosslinked PMCL was cut into 16 mm × 20 mm × 2 mm strips. Mechanical properties were measured by cyclic stretch of 10% strain over 100 cycles (stretching speed: 10 mm/min) using an Instron 5540A universal testing machine.

ICarP with different insertion depth of optical fiber was pre-adhered onto one end of myocardium using CCS@gel fixed in the wedge grip G1601-3 from MARK-10, the other end of the myocardium and the tapered optical fiber (TOF) were connected to M5I digital dynamometer from MARK-10. Adhesion strength between the patch substrate and TOF was obtained by measuring the force required to detach optical fiber from the patch.

Supplementary Note 3. Patch substrate degradation in vitro.

Discs of crosslinked PMCL (8 mm diameter) were incubated in PBS or PBS containing 2% Lipase B (w/w) at 37 °C, shook at 100 rpm (n = 4). Sample weight was recorded every 5 days.

Supplementary Note 4. Illumination demonstration on rat liver and hindlimb muscles.

Rat abdominal cavity was opened to expose the liver, then iCarP was adhered onto the liver by CCS@gel. After 5 min 660 nm illumination, the abdominal cavity was sutured, and TOF was removed using the similar method described in the myocardial infarction model. In the hindlimb muscle model, the thigh skin was incised to expose gracillis and flexor digitorum superficialis, followed by

attachment of iCarP using CCS@gel. The hindlimb was stretched to evaluate the robustness of tissue adhesion and the adhered, on-state iCarP. After laser illumination, the hindlimb skin was sutured, and TOF was removed.

Supplementary Note 5. Demonstration of 445 nm laser transmission.

Laser diodes with 445 nm wavelength (LSR445NL) from Lasever Inc. was used as light sources, output power of laser diode or illumination intermittent were adjusted by controlling the pump current of laser diode drivers. Illumination on ex vivo porcine heart was recorded by a camera.

Supplementary Note 6. Tumor photodynamic therapy in mice.

Nude mice (12 weeks old) were inoculated with 1×10^6 4T1 cells into the breast pad, and randomly divided into six groups: (1) Control; (2) FITC; (3) FEOF; (4) iCarP; (5) FEOF + FITC; (6) iCarP + FITC. For groups 2, 5 and 6, 50 μL of FITC (0.1 mg/mL) were injected into the tumors. Solid-state laser with wavelengths of 473 nm (CF60070, Changchun New Industries Optoelectronics Tech, China) were used as light sources for FEOF and iCarP (100 mW). ICarP indwelled in mice, animals received daily FITC injection and illumination for 3 days. Tumor volume and mouse body weight were monitored during the whole treatment (14 days). Tumor volume V (mm^3) = length × $width^2/2$. Fourteen days after initial illumination, tumor tissues were excised and fixed in 5% formaldehyde. Tissue sections with a thickness of 5 μm were stained with hematoxylin and eosin (H&E), CD31and TUNEL. All sections were examined by a virtual slide microscopy (Olympus VS200, USA).

Supplementary Note 7. Characterization of light field distribution of different optical fibers.

Flat-end optical fiber and tapered optical fibers with core diameter of 62.5 μm, clinically used side glow optical fiber (core diameter: 400 μm, diffusion length: 20 mm), clinically used matt flat-end optical fiber (core diameter: 400 μm) were successively fixed on the center of a rotating breadboard (Thorlabs, USA) and remained stationary during the test. An optical power meter was fixed on the edge of the round breadboard and adjusted to the same height of the optical fibers. By rotating the round breadboard, the optical power meter revolved around the tips of these fibers and detected the output power at different angles (-90° to 90°). Considering that all these fibers are axially symmetric, the optical power distribution in the horizontal plane could represent the spatial distribution in the spherical space.

Supplementary Note 8. Intramyocardial injection of Chlorella.

Male SD rats were anesthetized with 4% chloral hydrate by intraperitoneal injection, followed by endotracheal intubation and assisted ventilation. The rats were placed in the supine position, followed by a left thoracotomy and pericardectomy to expose the hearts. Chlorella (100 uL, 2×10^7/mL) suspension was injected into LV wall of rats, followed by swab pressing for hemostasis. Subsequent to iCarP implantation, chest cavity, muscles and skin were sutured with 3-0 silk sutures. Rats were anesthetized and sacrificed at 3 d post injection to harvest Chlorella-injected hearts. Rat hearts were fixed with 4% paraformaldehyde, embedded in paraffin, and sectioned, followed by immunochemistry stained with IL-6 and TNF-α to evaluate LV inflammation. Stained slides were observed using an Olympus IX51 microscope. Images were captured using DP2-BSW software (Olympus, VS200, USA).

Supplementary Note 9. Immunofluorescent staining

Rats were anesthetized and sacrificed at 1 d post MI to harvest treated hearts. Rat hearts were fixed with 4% paraformaldehyde, embedded in paraffin, and sectioned. The hearts were immunofluorescently stained with DAPI, cTnT and Bcl-2 (Abcepta, China) /Bax (Abcam, UK) /Cleaved Caspase-3 (CST, US) to reveal apoptosis. Images were captured using DP2-BSW software (Olympus, VS200, USA), and analyzed by ImageJ software.

Supplementary Note 10. RNA-seq analysis.

RNA-seq analysis was performed according to the previous method[2]. Rats were anesthetized and sacrificed at 1 d post MI to harvest treated hearts of Sham, MI, and iCarP+/Light+ groups, followed by excising and storing at -80 °C before analysis (n = 4). RNA-seq experiments were performed by Novogene (Beijing, China). According to the manufacturer's recommendations, sequencing libraries were generated using NEBNext UltraTM RNA Library Prep Kit for Illumina (NEB, USA), and index codes were added to attribute sequences to each sample. Following the manufacturer's instructions, TruSeq PE Cluster Kit v3-cBot-HS (Illumina, USA) was used for the clustering of the index-coded samples on a cBot Cluster Generation System. After cluster generation, the libraries preparations were sequenced on an Illumina Novaseq 6000 platform, and 150 bp paired-end reads were generated. For data analysis, the short reads (Raw data) were transformed from original fluorescence image files obtained from the Illumina platform and recorded in FASTQ format, which contains sequence information and corresponding sequencing quality information. We used Fastp (version 0.19.7) to perform basic statistics on the quality of the raw reads. Clean data (clean reads) were obtained by

removing reads containing adapter contamination, low-quality nucleotides, and unrecognizable nucleotide (N) from Raw data. The reference genome and gene model notes files were downloaded directly from genome website. RNA-seq data were analyzed using Hisat2[3] and featureCounts[4] with DESeq2[5] to elucidate differentially expressed genes (adjusted p value < 0.05 and Log2 (fold change) > 1 or < −1). Gene ontology (GO) analyses were performed by the Database for Annotation, Visualization, and Integrated Discovery[6] (DAVID). Transcriptomic data from bulk RNA sequencing are available through the National Center for Biotechnology Information Gene Expression Omnibus (GEO) under series accession no. GSE223691. Code is available upon request.

Supplementary Note 11. ROS accumulation in cardiomyocytes after Chlorella and illumination treatment.

H9C2 cells (Cell Bank of Typical Culture Collection of Chinese Academy of Sciences, China) were divided into 4 groups in random: (1) Chlorella-/Light- (without Chlorella injection and light treatment); (2) Chlorella-/Light+ group (without Chlorella injection but with light treatment); (3) Chlorella+/Light- group (with Chlorella injection but without light treatment); (4) Chlorella+/Light+ group (with Chlorella injection and light treatment), followed by seeding at a density of 5×10^3 in DMEM with 10% FBS. After 24 h, the medium was replaced with fresh DMEM with 10% FBS in Chlorella-/Light- and Chlorella-/Light+ group, and fresh DMEM with 10% FBS and 10^6/mL Chlorella in Chlorella+/Light- and Chlorella+/Light+ group, respectively. Chlorella-/Light- and Chlorella+/Light- group were incubated in dark at 37 °C and 5% CO_2, as Chlorella-/Light+ and Chlorella+/Light+ group were incubated in light (660 nm, 55 mw) at 37 °C and 5% CO_2. After 3 h, DCFH-DA (1:2000 v/v, in DMEM) was added. After 30 min, the wells were washed 3 times with PBS to remove excess probes. The fluorescence at 525 nm was excited by 488 nm, followed by measuring by a microplate reader (TECAN INFINITE M200PRO).

Supplementary Note 12. Phototoxicity assay.

The H9C2 cells was seeded at a density of 5×10^3 in DMEM with 10% FBS. After 24 h, the medium was replaced with fresh DMEM with 10% FBS and 10^6/mL Chlorella, followed by incubation with different duration in light at 37 °C and 5% CO_2. After incubation, the medium was replaced with DMEM containing 10% v/v CCK-8 reagent. After 2 h incubation, the absorbance of the medium at 450 nm was measured by a microplate reader (TECAN INFINITE M200PRO).

Supplementary Note 13. Proliferation assay of cardiac fibroblasts.

Cardiac fibroblasts (CFs) were isolated from 2 to 3 days old neonatal rats using enzyme digestion method. Then CFs were divided into 4 groups in random: (1) Chlorella-/Light- (without Chlorella injection and light treatment); (2) Chlorella-/Light+ group (without Chlorella injection but with light treatment); (3) Chlorella+/Light- group (with Chlorella injection but without light treatment); (4) Chlorella+/Light+ group (with Chlorella injection and light treatment), followed by seeding at a density of 5×10^3 in DMEM with 10% FBS. After 24 h, the medium was replaced with fresh DMEM with 10% FBS in Chlorella-/Light- and Chlorella-/Light+ group, and fresh DMEM with 10% FBS and 10^6/mL Chlorella in Chlorella+/Light- and Chlorella+/Light+ group, respectively. Chlorella-/Light- and Chlorella+/Light- group were incubated for 3 h in dark at 37 °C and 5% CO_2, as Chlorella-/Light+ and Chlorella+/Light+ group were incubated in light for 3 h (660 nm, 55 mw) at 37 °C and 5% CO_2. After light treatment, the CFs were continuedly incubated at 37 °C and 5% CO_2 for 1, 3 and 5 days. After the rated incubation time, the medium was replaced with DMEM containing 10% v/v CCK-8 reagent. After 2 h incubation, the absorbance of the medium at 450 nm was measured by a microplate reader (TECAN INFINITE M200PRO).

Supplementary Note 14. Electrophysiology and myocardial contractility assay in vitro.

Primary cardiomyocytes were isolated from 2 to 3 days old neonatal rats using enzyme digestion method. Then cells were divided into 2 groups in random: (1) Chlorella+ group (with Chlorella injection); (2) Chlorella- (without Chlorella injection), followed by culturing in DMEM with 10% FBS in E-Plate Cardio 96, which had microelectrodes (MEs) and interdigitated electrodes (IDEs). The extracellular action potential (EAP) was recorded by MEs. When primary cardiomyocytes produced rhythmic contraction, it caused changes in cell morphology. Increasing the excitation frequency of IDEs could simultaneously obtain mechanical beating (MB) of the cells. At day 5, light treatment (660 nm, 55 mW) was performed in both groups for 3 h. The electrophysiology and beating of the cells through the laser and xCELLigence RTCA Cardiosystem system (Agilent Technologies Inc, USA) were collected. Feature parameters including EAP amplitude, MB amplitude and firing rate were extracted for analysis to quantify the characteristics of the EAP and MB.

Supplementary Note 15. Measurement of heart rate of iCarP treated dog.

The procedure of preoperative preparation and anesthesia was the same as described in the "minimally invasive implantation" section in Methods. Lactated Ringer was used to replenish body fluids. The dog was adjusted to supine posture on heated operation table, the thoracic surgery area was shaved, cleaned, disinfected, and aseptic hole-towel was laid. An incision was made between the fifth rib and the sixth rib, the skin and muscle are cut in sequence, the pleura was opened, and the thoracic

retractor was used to expand the surgical field. After confirming the position of the heart, the pericardium was cut to expose the surface of the heart. ICarP was attached to the apex of the heart for illumination, and iCarP was detached after recording the electrocardiography data as baseline. Subsequently, LAD and LCx was ligated to cause myocardial infarction, and iCarP was again attached to repeat above process, recording the electrocardiography data. After the surgery, iCarP was detached and the pericardium was sutured. The instruments were taken out, the chest cavity was sutured and closed, disinfected with povidone iodine, and gauze was applied to cover the wound. Ventilator was retained for 1 hour before recovery of spontaneous respiration. For analgesia and anti-inflammation, 0.6 mL meloxicam was subcutaneously injected.

$$F(z) = 62.5 - 56.36e^{-\left(\frac{0.485z+16.18}{65.81}\right)^2} + 0.6324e^{-\left(\frac{0.485z+0.5469}{0.1508}\right)^2} + 1.103e^{-\left(\frac{0.485z+24.78}{-0.02043}\right)^2} - 12.1e^{-\left(\frac{0.485z+13.07}{-22.32}\right)^2}$$

Supplementary Equation 1. Shape of the tapered optical fiber. F(z) is the radius of TOF at position z (**Fig. 2f**).

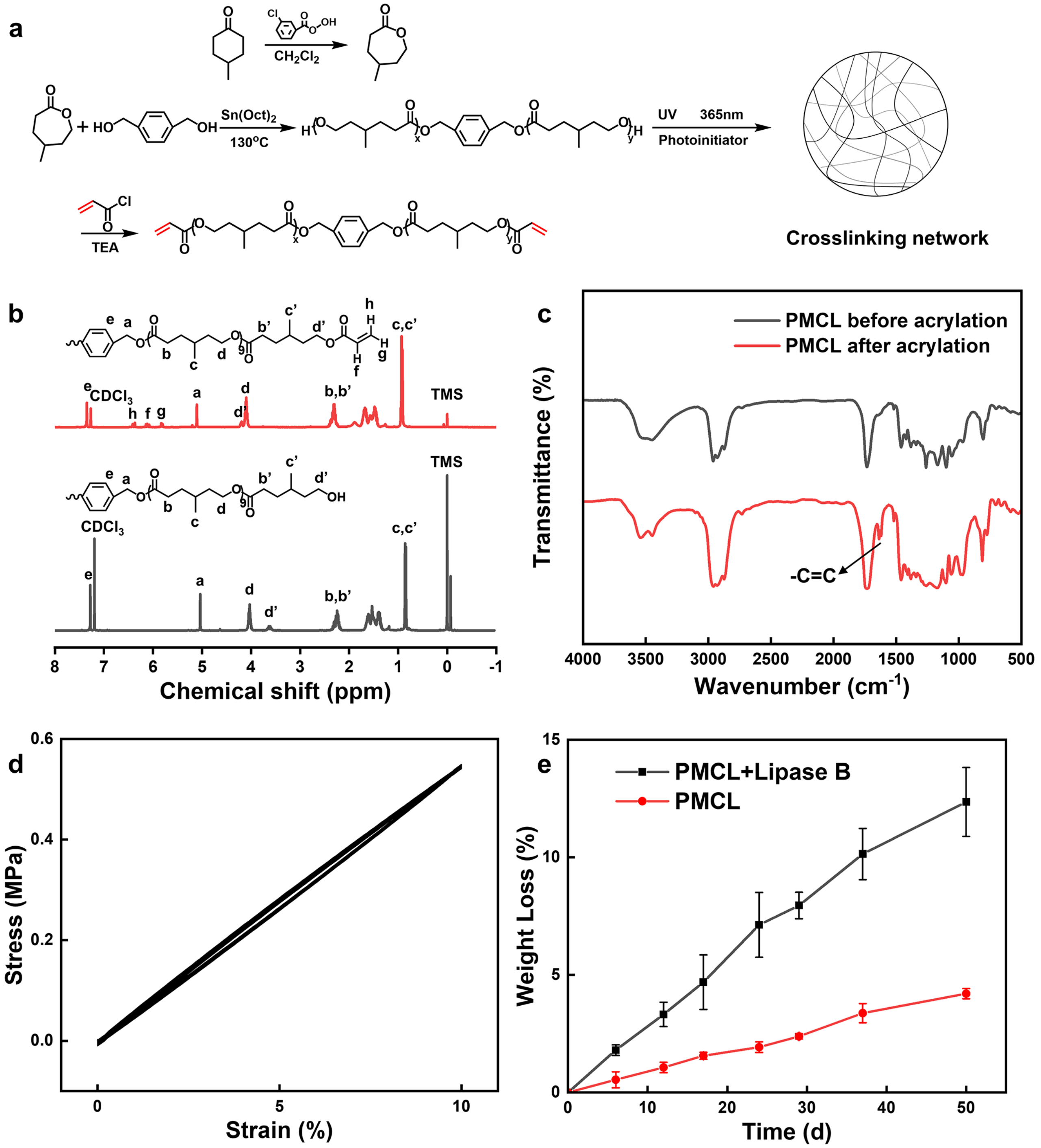

Supplementary Figure 1. Fabrication and characterization of PMCL patch. **a** Synthesis (left) and crosslinking of PMCL patch (right). **b** The structure and ^{1}H NMR spectra of PMCL after acrylation (up) and PMCL before acrylation (down). **c** FT-IR of PMCL before (black) and after di-acrylation (red). **d** Cyclic stretch of PMCL patch at 10% strain for 100 times. **e** Degradation of PMCL patches with or without Lipase in vitro (n=3 per group), data are presented as means ± SD.

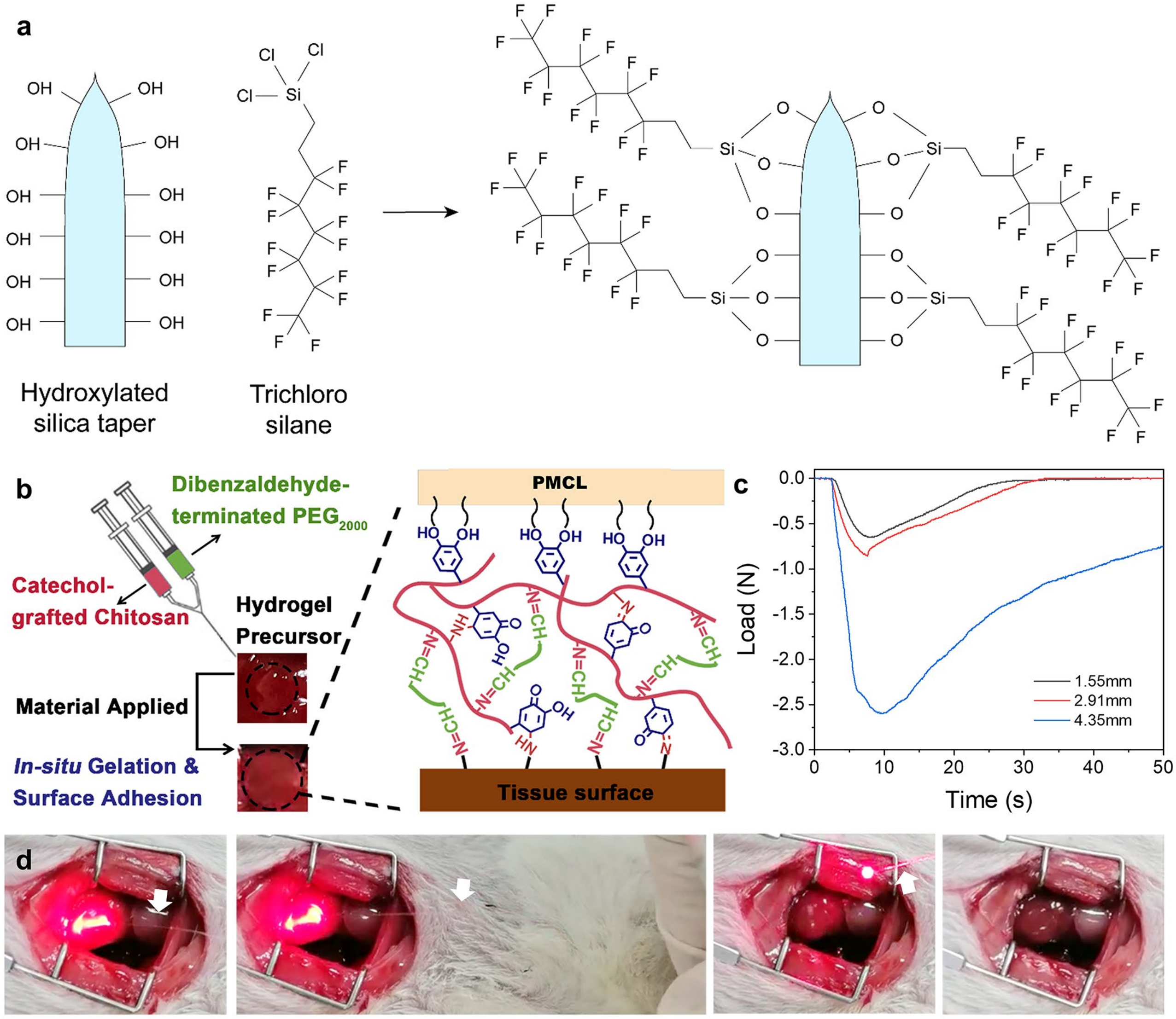

Supplementary Figure 2. ICarP adhesion and TOF removal. **a** Scheme of trichloro silane treatment of TOF surface. **b** Scheme and device adhesion on tissue surface by CCS@gel[1]. **c** Adhesion strengths between PMCL substrate and TOF at different insertion depths. Mechanical loads during pulling-out of TOFs from PMCL patch. **d** Removal of TOF from an iCarP adhered on a beating rat heart using the same process in Fig. 5b.

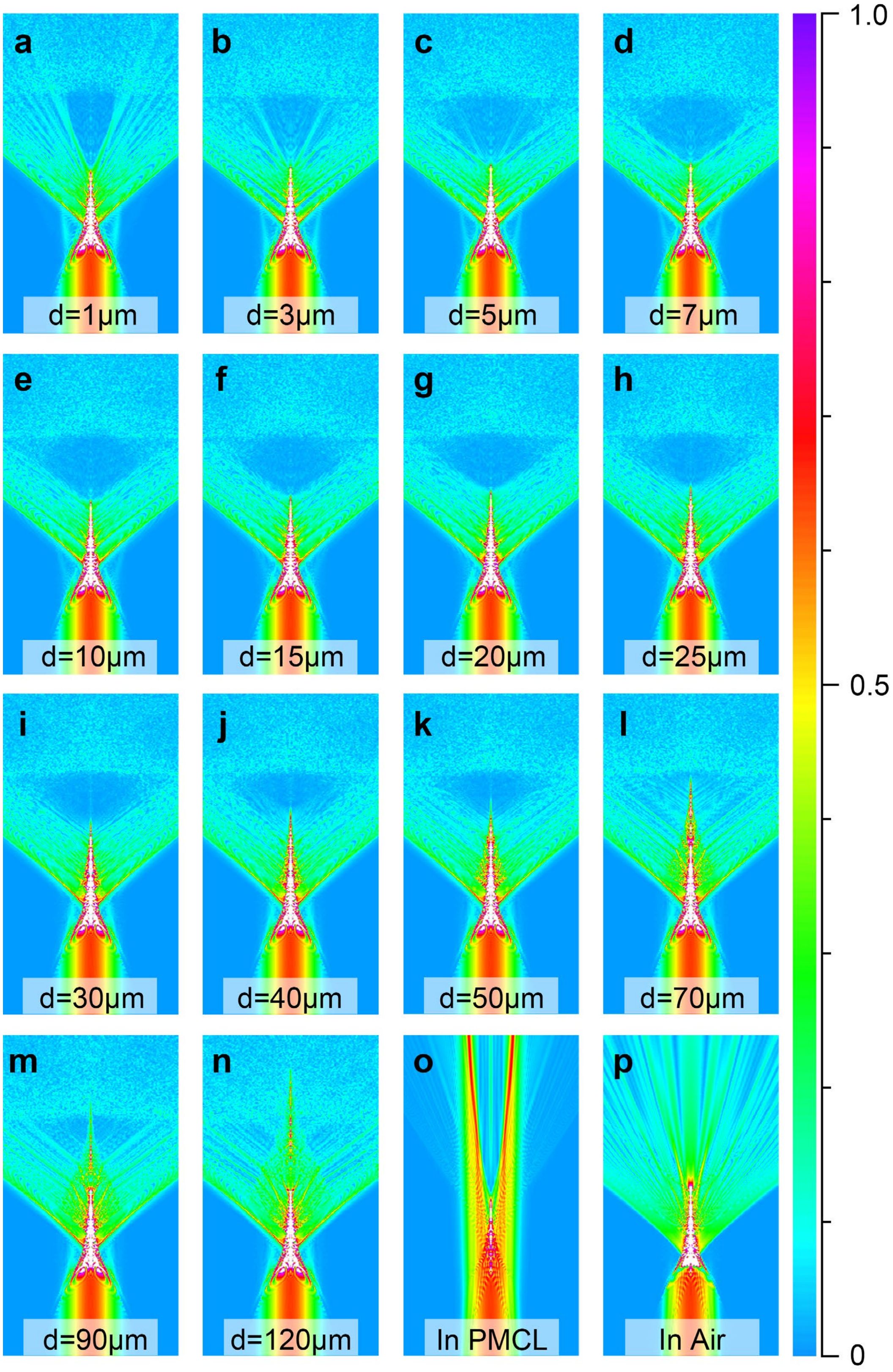

Supplementary Figure 3. Electric field intensity distribution of light emitting for iCarP with different distance between the TOF tip and air gap tip (*d*). a-n 1-120 μm. **o** 0 μm (TOF in PMCL without air gap). **p** TOF in air without PMCL.

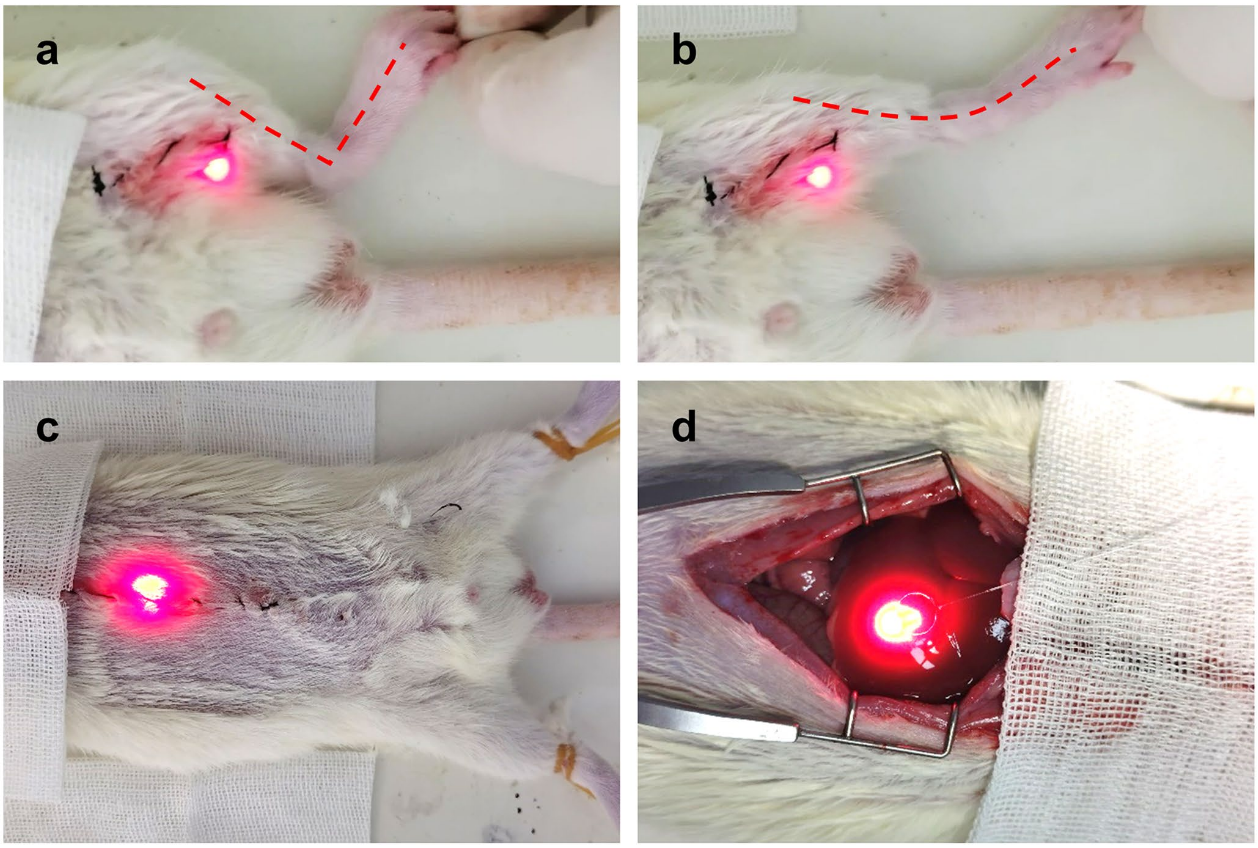

Supplementary Figure 4. Demonstration of iCarP application on rat hindlimb muscle and liver. ICarP illumination on (**a**) bent, (**b**) stretched hindlimb (the red dotted line represents the hindlimb of rat) and liver in (**c**) closed, (**d**) opened abdominal cavity.

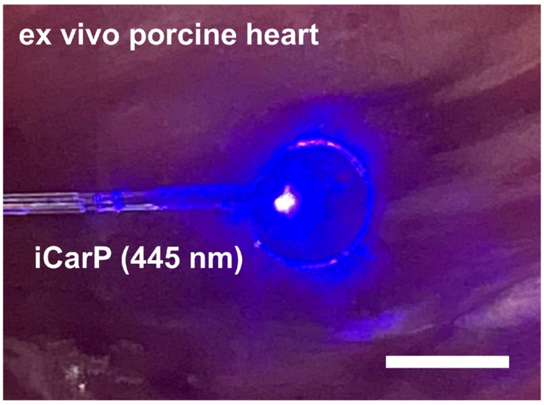

Supplementary Figure 5. ICarP guided light with wavelength of 445 nm onto an ex vivo porcine heart. Scale bar = 5 mm.

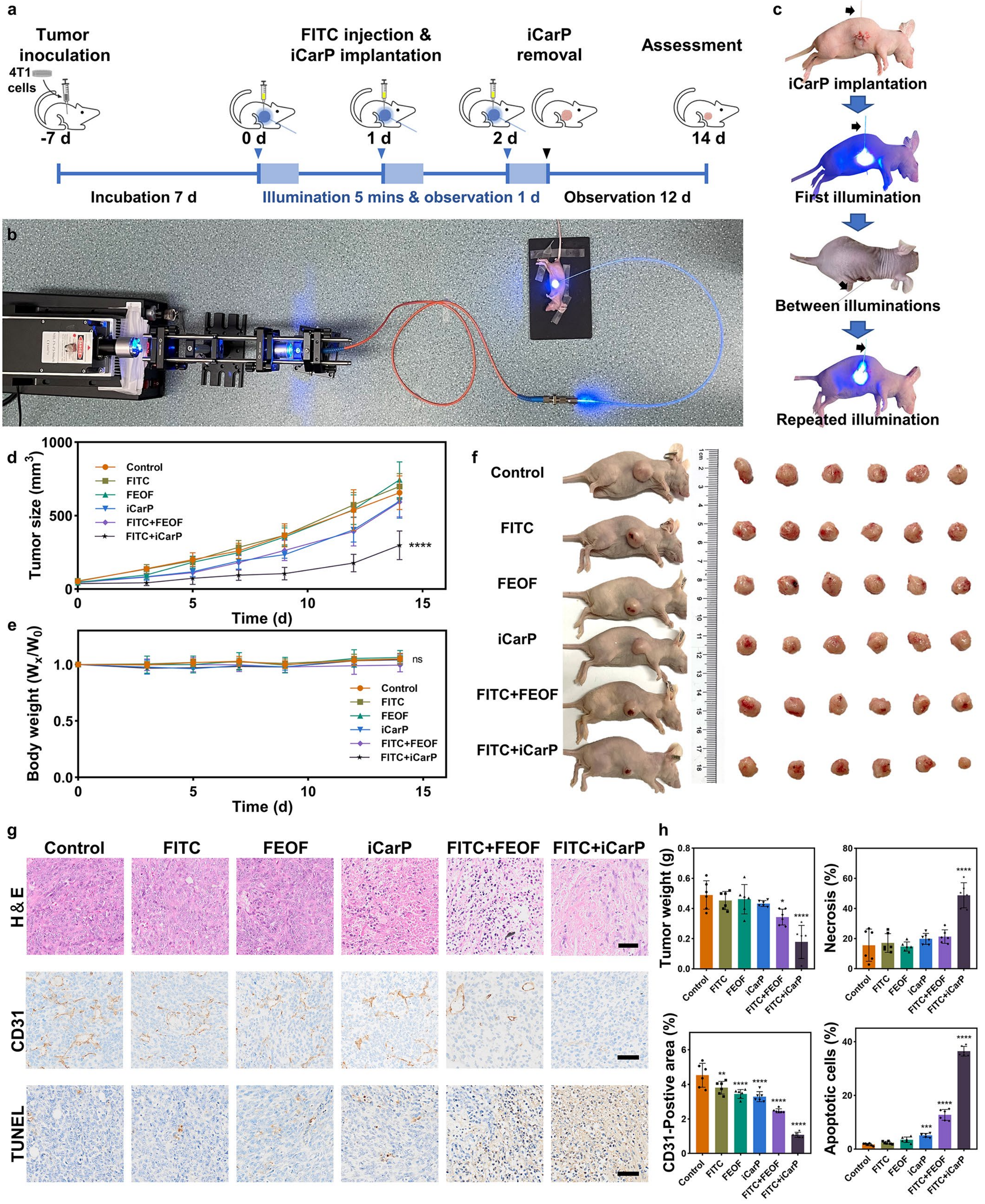

Supplementary Figure 6. Effects of tumor photodynamic therapy in mice. a Timeline of the animal study. Mice received following treatments 7 days after 4T1 cells inoculation. Control group: no treatment; FITC group: FITC injection in tumor, no illumination; FEOF group: flat-end optical fiber

illumination without FITC injection; iCarP group: iCarP illumination without FITC injection; FITC+FEOF group: FITC injection with FEOF illumination; FITC+iCarP group: FITC injection with repeated iCarP illumination. **b** Image of the mice and equipments during iCarP illumination. **c** Photos of repeated iCarP illumination on the same mouse. **d** Growth curves of tumor after different treatments (n = 6 per group). **e** Body weight curves of mice for 14 d after different treatments (n = 6 per group). Statistical significance was calculated using one-way ANOVA with Bonferroni's posttest to compare Tumor size and Body weight on d 14 among the 6 groups and data are presented as means ± SD, ****$p < 0.0001$ vs control group. **f** Photos of mice and the excised tumors 14 d after treatment**. g** Representative H&E, CD31, and TUNEL staining images of tumors, scale bar = 50 μm. **h** Quantitative analysis of tumor weight, necrosis (%), CD31 positive area (%), and apoptotic cells (%) (n = 6 per group). Statistical significance was calculated using one-way ANOVA with Bonferroni's posttest and data are presented as means ± SD. *$p < 0.05$, **$p < 0.01$, ***$p < 0.001$, ****$p < 0.0001$ vs control group. Source data and exact *p*-values are provided in the Source Data file.

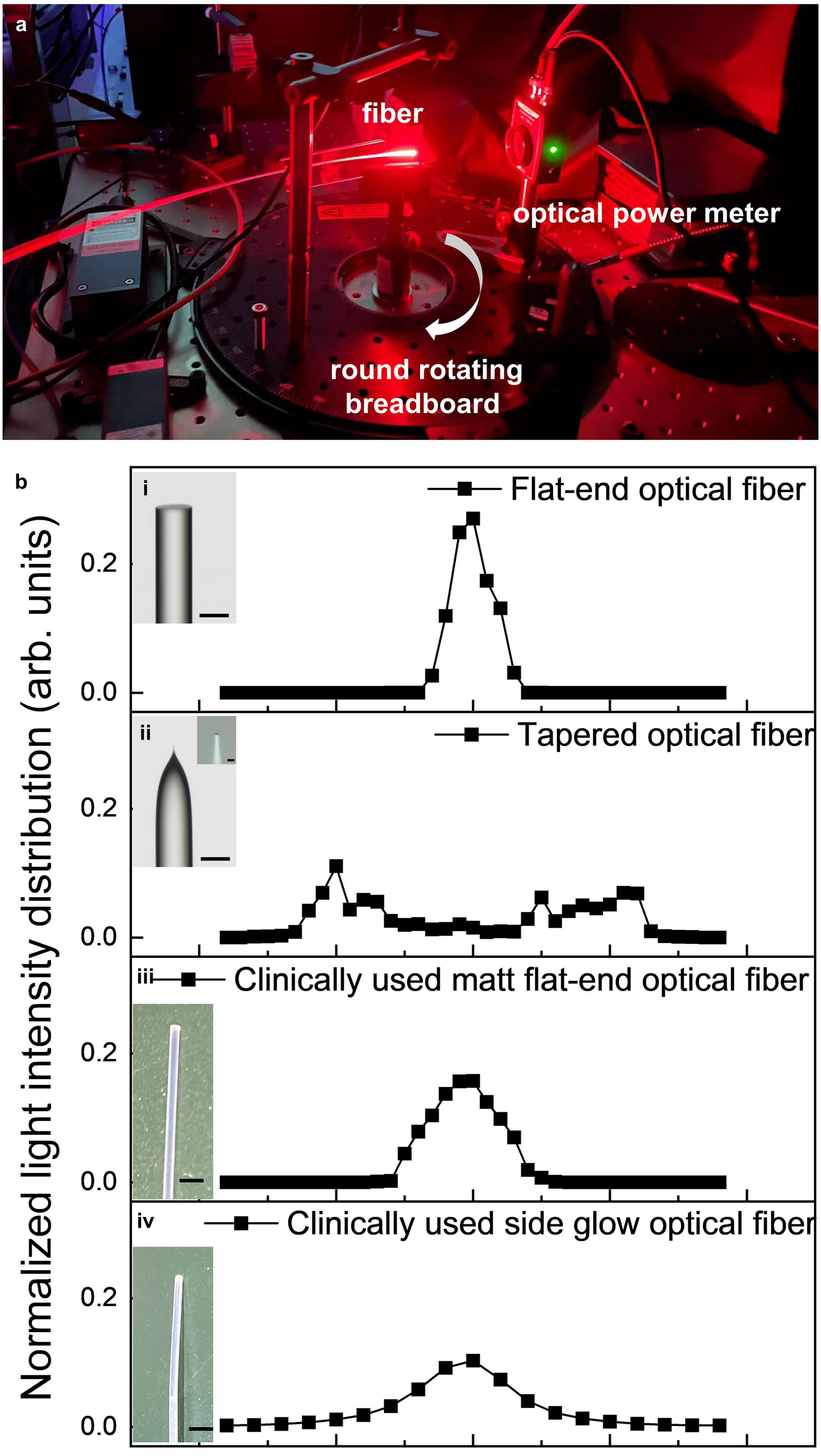
a
fiber
optical power meter
round rotating
breadboard
b
Flat-end optical fiber
Tapered optical fiber
Clinically used matt flat-end optical fiber
Clinically used side glow optical fiber
i
ii
iii
iv
Normalized light intensity distribution (arb. units)
0.0
0.2
-100
-50
0
50
100
Angle (°)

Supplementary Figure 7. Light scattering effects of flat-end optical fibers, tapered optical fibers, and clinically used optical fibers. a Equipment for light distribution measurement. **b** Normalized light distribution of (i) flat-end optical fiber (core diameter: 62.5 μm), inset: microscopic view of the tip of flat end, scale bar = 100 μm. (ii) Tapered optical fiber, inset: microscopic view of tapered fiber end, scale bar = 100 μm. (iii) Clinically used matt flat-end optical fiber (core diameter: 400 μm), inset: appearance of fiber end, scale bar = 4 mm. (iv) Clinically used side glow optical fiber, inset: appearance of the fiber end, scale bar = 4 mm. Insets of i-iv are representative images of ≥2 samples.

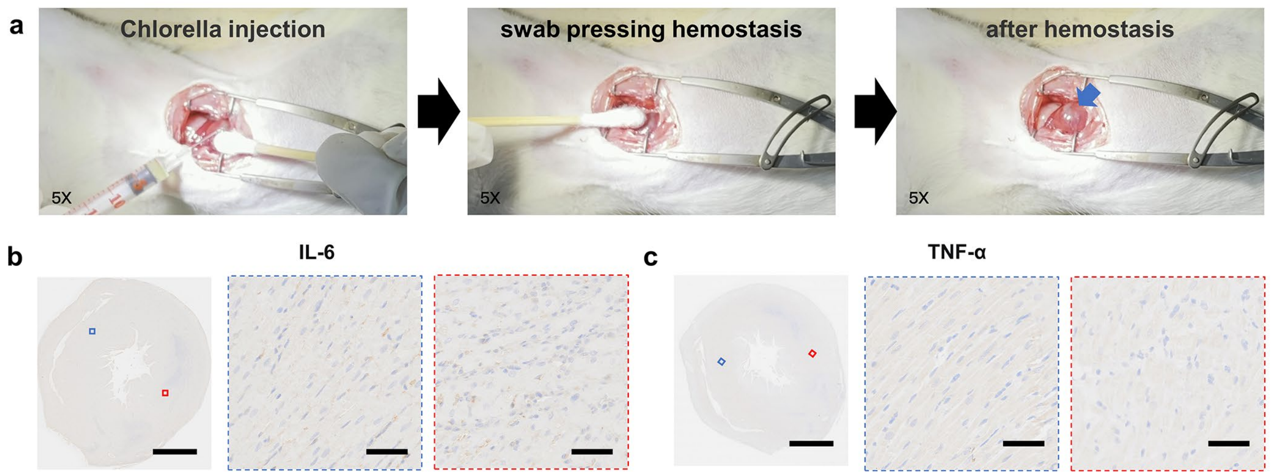

Supplementary Figure 8. Evaluation of the potential risks of bleeding and inflammation caused by Chlorella injection. a Chlorella suspension was injected into LV wall of rats, followed by swab pressing for hemostasis (injection site and distributed Chlorella are pointed by blue arrow). Minor bleeding caused by the insulin needle was stopped by swab pressing within seconds. Corresponding video was added as Supplementary video 6. **b, c** Representative immunofluorescent staining images of IL-6 (**b**) and TNF-α (**c**) in rat hearts 3 d after Chlorella injection in LV myocardium, n = 3 biologically independent samples, scale bar = 2 mm. Inset: magnified images, scale bar = 50 µm. Each experiment was repeated 4 times independently.

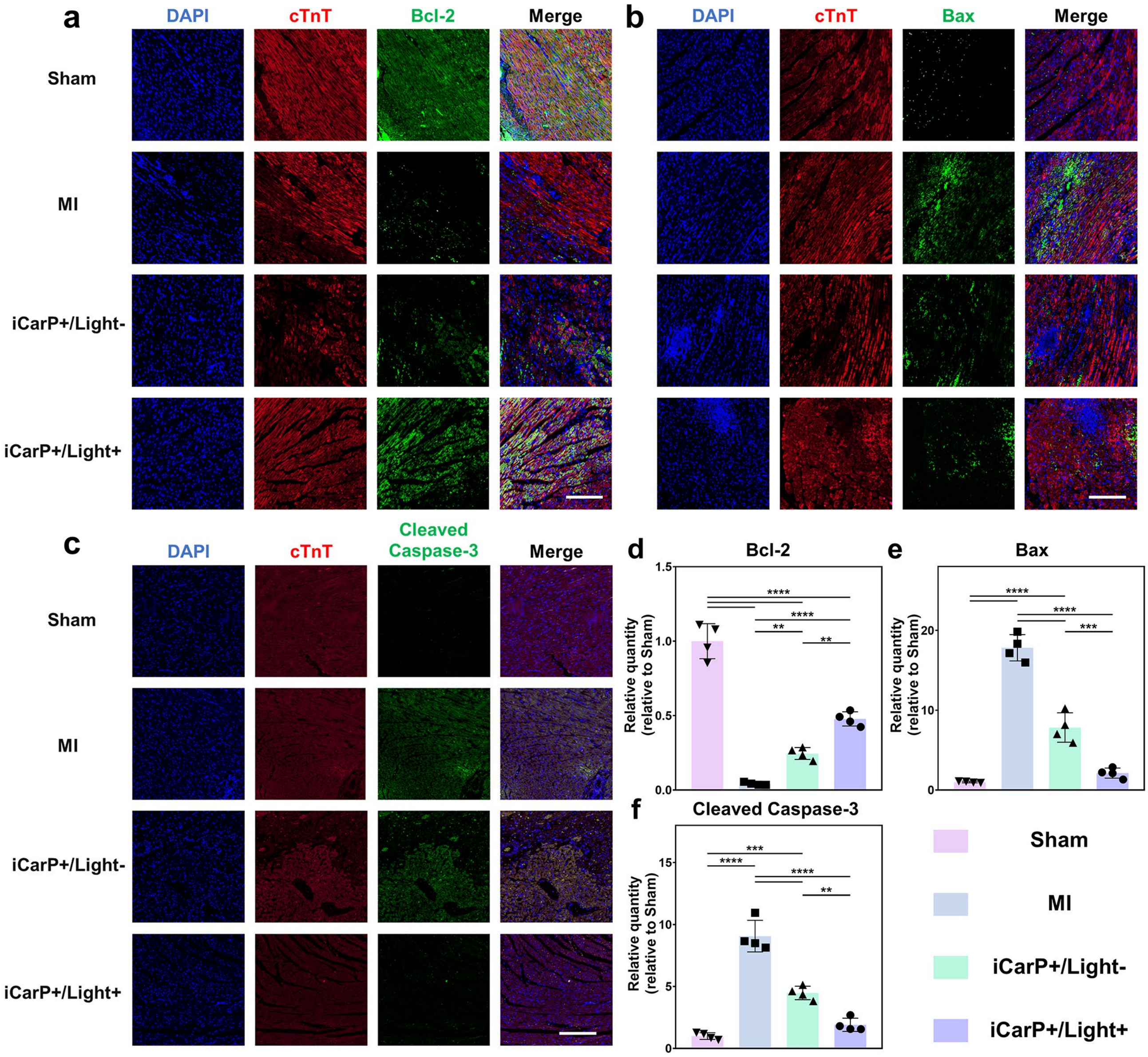

Supplementary Figure 9. Expression of apoptosis markers in myocardium. Representative immunofluorescent staining images of cTnT/Bcl-2 **(a)**, cTnT/Bax **(b)** and cTnT/Cleaved Caspase-3 **(c)** in left ventricular 1 d after MI (nuclei: blue, cTnT: red, Bcl-2/Bax/Cleaved Caspase-3: green). Scale bars = 200 μm. Quantitative analysis of expression of Bcl-2 **(d)**, Bax **(e)** and Cleaved Caspase-3 **(f)** (n = 4 per group). Statistical significance was calculated using one-way ANOVA with Tukey's posttest and data are presented as means ± SD. $^{**}p < 0.01$, $^{***}p < 0.001$, $^{****}p < 0.0001$. Source data and *p*-values are provided in the Source Data file.

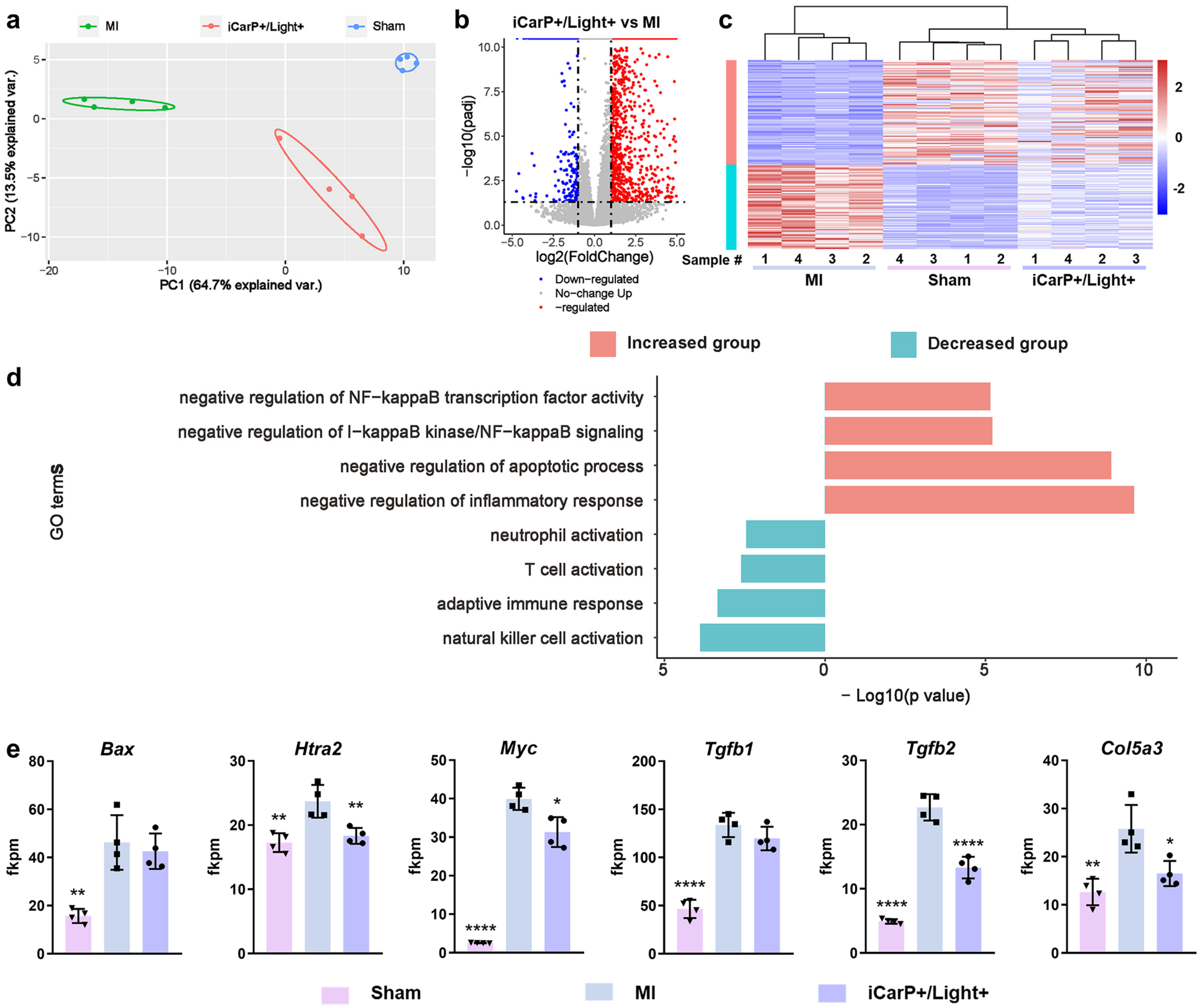

Supplementary Figure 10. RNA-seq analysis of cardiac tissues 1 day after surgery. a Principal component analysis (PCA) of transcriptomes of LV myocardium from MI, iCarP+/Light+ and Sham groups (n = 4 per group). **b** Volcano plot of all expressed genes from the iCarP+/Light+ and MI group (n = 4 per group). The vertical dashed lines represent the border of gene expressions in the iCarP+/Light+ group greater than 1-fold change vs the MI group. Dots above the horizontal dashed line have adjusted $p < 0.05$, which was calculated using DESeq2 with Benjamini–Hochberg correction (two-sided). **c** Clustering analysis of the Sham, MI, and iCarP+/Light+ groups using apoptosis-related genes (Increased group, up-regulated genes in Sham and iCarP+/Light+ groups compared to in MI group; Decreased group, down-regulated genes in Sham and iCarP+/Light+ groups compared to in MI group; n = 4 per group). **d** Gene Ontology (GO) Biological Process (BP) analyses of iCarP effective genes. Statistically enriched biological process annotations were identified through the online DAVID bioinformatics resource (https://david.ncifcrf.gov/summary.jsp). One-tail Fisher Exact *p* value were determined for the GO term analysis. **e** Expression levels of *Bax, Htra2, Myc, Tgfb1, Tgfb2* and

Col5a3 genes in Sham, MI, and iCarP+/Light+ groups (n = 4 per group). Statistical significance was calculated using two-tailed unpaired t-test and data are presented as means ± SD. $*p < 0.05$, $**p < 0.01$, $****p < 0.0001$ vs MI group. Source data and *p*-values are provided in the Source Data file.

Principal component analysis (PCA), volcano graph (Log2(fold change) > 1 or < −1, adjusted p value < 0.05) and clustering analysis shown in Supplementary Fig. 10a-c, demonstrated that iCarP triggered in situ photosynthesis significantly altered the transcriptome compared to MI group, and recovered the gene expressions to levels close to those in the Sham group. Gene Ontology (GO) Biological Process (BP) analysis revealed that up-regulated genes in the Sham and iCarP+/Light+ group compared to the MI group were involved in negative regulation of NF-kappaB transcription factor activity, negative regulation of I-kappaB kinase/NF-kappaB signaling, negative regulation of apoptotic process and negative regulation of inflammatory response, indicating that in situ photosynthesis could protect the LV myocardium against MI by reducing apoptosis and inflammation, which is consistent with the histological results in Fig. 5. The down-regulated genes were involved in neutrophil activation, T cell activation, adaptive immune response and natural killer cell activation, supporting that in situ photosynthesis could reduce inflammation (Supplementary Fig. 10d).

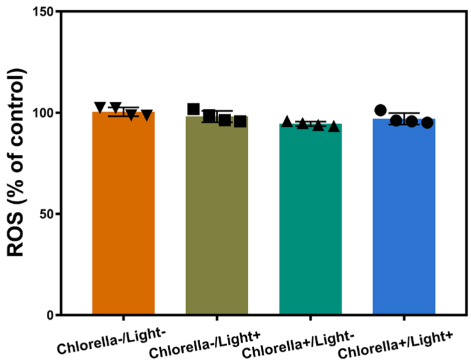

Supplementary Figure 11. Effect of Chlorella and light on ROS accumulation in cardiomyocytes. Cells were stained by DCFH-DA (n = 4 per group), data are presented as means ± SD.

352

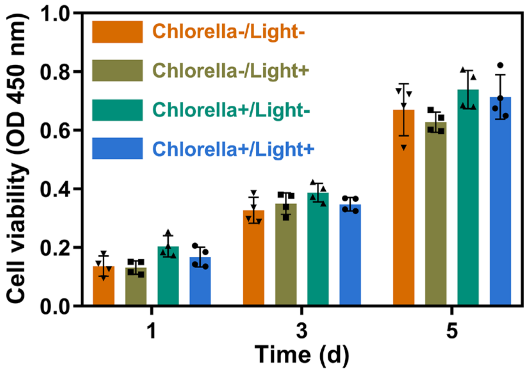

353
354 **Supplementary Figure 12. Effect of Chlorella and light on cardiac fibroblasts proliferation.** Data
355 are presented as means ± SD, n = 4 per group.
356

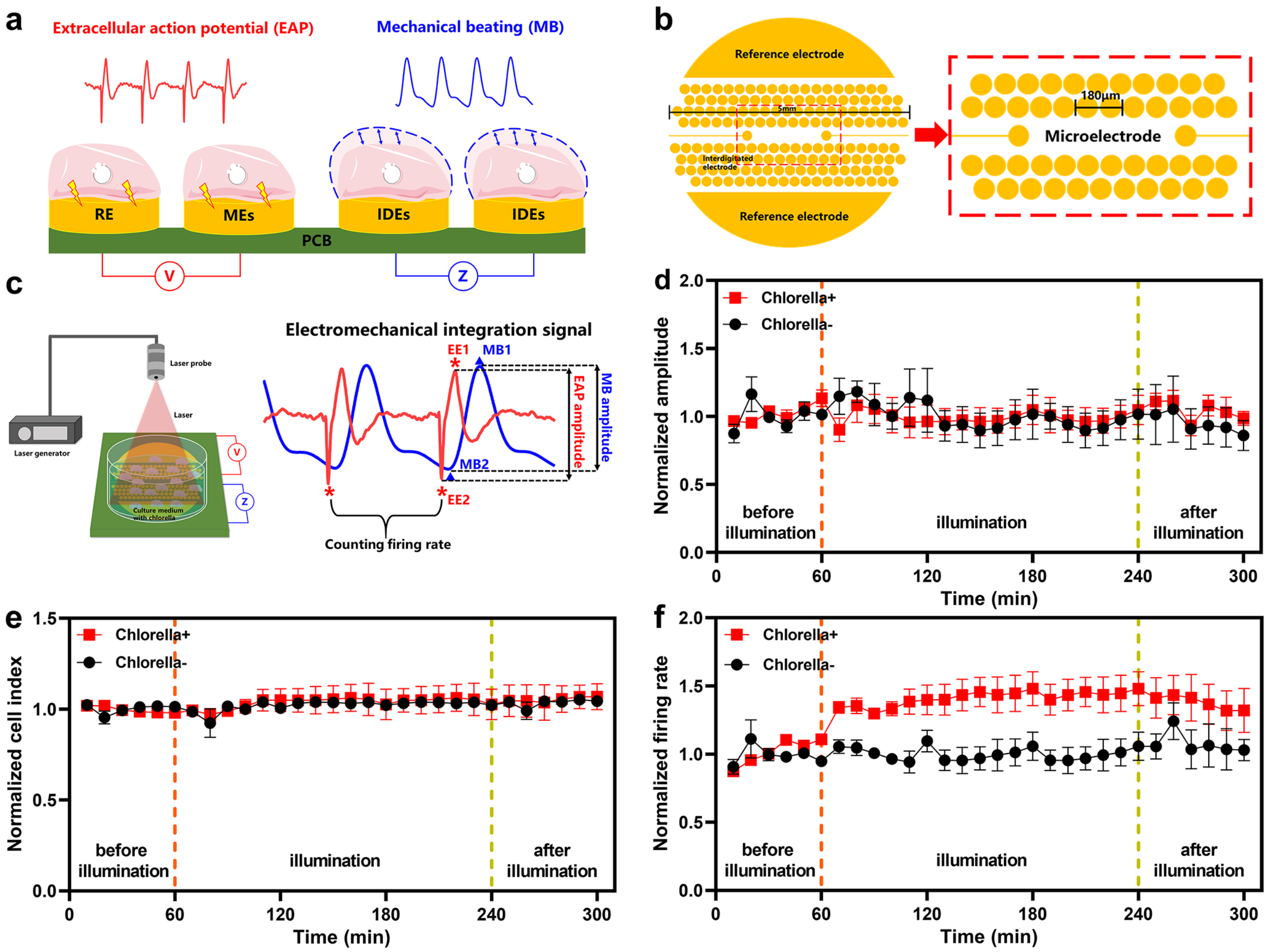

Supplementary Figure 13. Effects of Chlorella and light on myocardial electrophysiology and myocardial contractility in vitro. **a** Schematic diagram of the sensor for simultaneous detection of primary cardiomyocyte extracellular action potential and mechanical beating. **b** Electrode map of microelectrodes and interdigitated electrodes. **c** Schematic diagram of the experiment and definition of signal characteristic parameters. EE1 and EE2 were defined to label the peak, valley of extracellular action potential. MB1 and MB2 were defined to label the peak, valley of mechanical beating. **d-f** Effect of Chlorella and light on EAP amplitude (d), MB amplitude (e) and firing rate (f) in primary cardiomyocytes (n = 4 per group), data are presented as means ± SEM.

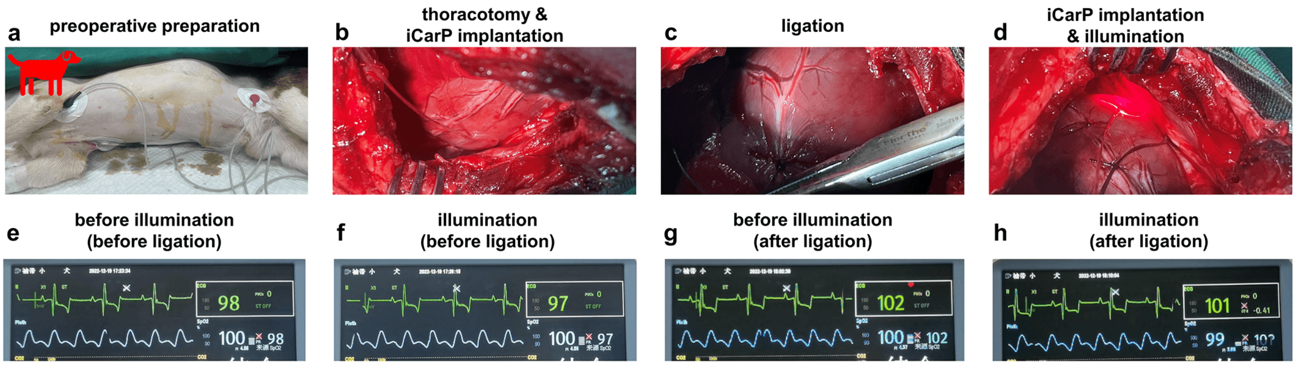

Supplementary Figure 14. Effects of iCarP on heart rate of a dog. a Preoperative preparation. **b** Thoracotomy & iCarP implantation. **c** Ligation of LAD and LCx. **d** ICarP implantation & illumination. **e-h** Dog heart rate measured by electrocardiogram at different stages of the surgery, including before illumination (before ligation) (**e**), during illumination (before ligation) (**f**), before illumination (after ligation) (**g**) and during illumination (after ligation) (**h**).

Supplementary Reference

1. Fang, W., Yang, L., Hong, L. & Hu, Q. A chitosan hydrogel sealant with self-contractile characteristic: From rapid and long-term hemorrhage control to wound closure and repair. *Carbohydr Polym* **271**, 118428 (2021).
2. Lu, Y., *et al.* A honeybee stinger-inspired self-interlocking microneedle patch and its application in myocardial infarction treatment. *Acta Biomater* **153**, 386-398 (2022).
3. Kim, D., Paggi, J.M., Park, C., Bennett, C. & Salzberg, S.L. Graph-based genome alignment and genotyping with HISAT2 and HISAT-genotype. *Nat Biotechnol* **37**, 907-915 (2019).
4. Liao, Y., Smyth, G.K. & Shi, W. featureCounts: an efficient general purpose program for assigning sequence reads to genomic features. *Bioinformatics* **30**, 923-930 (2014).
5. Love, M.I., Huber, W. & Anders, S. Moderated estimation of fold change and dispersion for RNA-seq data with DESeq2. *Genome Biol* **15**, 550 (2014).
6. Jiao, X., *et al.* DAVID-WS: a stateful web service to facilitate gene/protein list analysis. *Bioinformatics* **28**, 1805-1806 (2012).
