# Supplementary material for: A biodegradable, flexible photonic patch for in vivo phototherapy
Source: Nat Commun. 2023 May 27;14:3069. doi: 10.1038/s41467-023-38554-x (PMC10224912; doi:10.1038/s41467-023-38554-x)
Supplement: Supplementary file 3 — Description of additional supplementary files [file 41467_2023_38554_MOESM3_ESM.pdf]

Description of additional supplementary files

Supplementary Movie 1. iCarP supports different illumination wavelengths

Supplementary Movie 2. Pulsatile iCarP illumination at 1 Hz

Supplementary Movie 3. iCarP supports different illumination intensity

Supplementary Movie 4. Cardiac injection of Chlorella

Supplementary Movie 5. Removal of TOF after treatment while chest is closed

Supplementary Movie 6. iCarP implantation

Supplementary Movie 7. Effect of iCarP on heart rate in dog
