# Supplementary material for: A biodegradable, flexible photonic patch for in vivo phototherapy
Source: Nat Commun. 2023 May 27;14:3069. doi: 10.1038/s41467-023-38554-x (PMC10224912; doi:10.1038/s41467-023-38554-x)
Supplement: Supplementary file 11 — Reporting Summary [file 41467_2023_38554_MOESM11_ESM.pdf]

nature portfolio

Corresponding author(s): Yang Zhu

Last updated by author(s): Apr 28, 2023

Reporting Summary

Nature Portfolio wishes to improve the reproducibility of the work that we publish. This form provides structure for consistency and transparency in reporting. For further information on Nature Portfolio policies, see our Editorial Policies and the Editorial Policy Checklist.

Statistics

For all statistical analyses, confirm that the following items are present in the figure legend, table legend, main text, or Methods section.

n/a	Confirmed	
☐	☒	The exact sample size (*n*) for each experimental group/condition, given as a discrete number and unit of measurement
☐	☒	A statement on whether measurements were taken from distinct samples or whether the same sample was measured repeatedly
☐	☒	The statistical test(s) used AND whether they are one- or two-sided<br>*Only common tests should be described solely by name; describe more complex techniques in the Methods section.*
☒	☐	A description of all covariates tested
☐	☒	A description of any assumptions or corrections, such as tests of normality and adjustment for multiple comparisons
☐	☒	A full description of the statistical parameters including central tendency (e.g. means) or other basic estimates (e.g. regression coefficient) AND variation (e.g. standard deviation) or associated estimates of uncertainty (e.g. confidence intervals)
☐	☒	For null hypothesis testing, the test statistic (e.g. *F*, *t*, *r*) with confidence intervals, effect sizes, degrees of freedom and *P* value noted<br>*Give P values as exact values whenever suitable.*
☒	☐	For Bayesian analysis, information on the choice of priors and Markov chain Monte Carlo settings
☒	☐	For hierarchical and complex designs, identification of the appropriate level for tests and full reporting of outcomes
☒	☐	Estimates of effect sizes (e.g. Cohen's *d*, Pearson's *r*), indicating how they were calculated

Our web collection on statistics for biologists contains articles on many of the points above.

Software and code

Policy information about availability of computer code

Data collection	DP2-BSW software (Olympus, VS200, USA), SensorTrace v3.4.100.
Data analysis	Image-Pro Plus 6.0, GraphPad Prism 8.0.1, Origin 2018, GPower 3.1

For manuscripts utilizing custom algorithms or software that are central to the research but not yet described in published literature, software must be made available to editors and reviewers. We strongly encourage code deposition in a community repository (e.g. GitHub). See the Nature Portfolio guidelines for submitting code & software for further information.

Data

Policy information about availability of data

All manuscripts must include a data availability statement. This statement should provide the following information, where applicable:

- Accession codes, unique identifiers, or web links for publicly available datasets
- A description of any restrictions on data availability
- For clinical datasets or third party data, please ensure that the statement adheres to our policy

All relevant data related to this manuscript are available on request from the authors on reasonable request. RNA-Seq data were deposited at National Center for Biotechnology Information Gene Expression Omnibus (GEO) with accession number GSE223691 [https://www.ncbi.nlm.nih.gov/geo/query/acc.cgi?acc=GSE223691]. The remaining data are available within the Article, Supplementary Information or Source Data file. Source data are provided with this paper.

Human research participants

Policy information about studies involving human research participants and Sex and Gender in Research.

Reporting on sex and gender	No human research participants was involved in the study.
Population characteristics	No human research participants was involved in the study.
Recruitment	No human research participants was involved in the study.
Ethics oversight	No human research participants was involved in the study.

Note that full information on the approval of the study protocol must also be provided in the manuscript.

Field-specific reporting

Please select the one below that is the best fit for your research. If you are not sure, read the appropriate sections before making your selection.

☒ Life sciences ☐ Behavioural & social sciences ☐ Ecological, evolutionary & environmental sciences

For a reference copy of the document with all sections, see nature.com/documents/nr-reporting-summary-flat.pdf

Life sciences study design

All studies must disclose on these points even when the disclosure is negative.

Sample size	Before animal experiments, GPower was used to evaluate the sample size. Results showed that at least 4 samples per group were needed to reveal the differences. After the formal experiments, the data were analyzed, and verified that 4 per group was sufficient. At the same time, we also performed the normal distribution test and the homogeneity of variance test, the results met the requirements.
Data exclusions	No data were excluded from the analyses.
Replication	All in vitro and in vivo results are representative of two to six independents.
Randomization	All samples were randomly allocated into experimental groups.
Blinding	All data collection and analysis were blinded to group allocation.

Reporting for specific materials, systems and methods

We require information from authors about some types of materials, experimental systems and methods used in many studies. Here, indicate whether each material, system or method listed is relevant to your study. If you are not sure if a list item applies to your research, read the appropriate section before selecting a response.

Materials & experimental systems

n/a	Involved in the study	
☐	☒	Antibodies
☐	☒	Eukaryotic cell lines
☒	☐	Palaeontology and archaeology
☐	☒	Animals and other organisms
☒	☐	Clinical data
☒	☐	Dual use research of concern

Methods

n/a	Involved in the study	
☒	☐	ChIP-seq
☒	☐	Flow cytometry
☒	☐	MRI-based neuroimaging

Antibodies

Antibodies used	Anti-Connexin 43/GJA1 antibody - Intercellular Junction Marker (Acbam, ab11370, Polyclonal, 1:2000 dilution), Anti-Cardiac Troponin T antibody (Acbam, ab209813, EPR20266, 1:4000 dilution), Anti-Bcl-2 antibody (Abcepta, P10415, Polyclonal, 1:100 dilution), Anti-Bax antibody (Abcam, ab32503, E63, 1:250 dilution), Cleaved Caspase-3 Rabbit mAb(Cell Signaling Technology, #9664, 1:2000 dilution), Anti-IL6 antibodies (Affinity biosciences, DF6087, Polyclonal, 1:200 dilution), Anti-CD31 antibody (Abcam, ab182981, EPR17259, 1:2000 dilution), Anti-TNF-α Polyclonal antibody (ImmunoWay Biotechnology Company, YT4689, Polyclonal, 1:200 dilution)

Validation	Anti-Connexin 43 / GJA1 antibody - Intercellular Junction Marker (Acbam, ab11370), Anti-Cardiac Troponin T antibody (Acbam, ab209813), Anti-Bax antibody (Abcam, ab32503) and Anti-CD31 antibody (Abcam, ab182981) have been validated for use in immunohistochemistry , as stated on the Acbam product page; Anti-Bcl-2 Antibody (Abcepta, P10415) has been validated for use in immunohistochemistry , as stated on the Abcepta product page; Cleaved Caspase-3 Rabbit mAb (Cell Signaling Technology, #9664) has been validated for use in immunohistochemistry , as stated on the CST product page. Anti-TNF-α Polyclonal antibody (ImmunoWay Biotechnology Company, YT4689) has been validated for use in immunohistochemistry , as stated on the ImmunoWay Biotechnology Company product page.

Eukaryotic cell lines

Policy information about cell lines and Sex and Gender in Research

Cell line source(s)	4T1 murine breast cancer cells (ATCC), H9C2 cells (Cell Bank of Typical Culture Collection of Chinese Academy of Sciences)
Authentication	4T1 cell line and H9C2 cell line we used were morphologically confirmed according to the information provided by ATCC and Cell Bank of Typical Culture Collection of Chinese Academy of Sciences, respectively.
Mycoplasma contamination	4T1 cell line was tested for mycoplasma contamination. No mycoplasma contamination was found. H9C2 cell line was not tested for mycoplasma contamination.
Commonly misidentified lines (See ICLAC register)	No commonly misidentified cell lines were used in the study.

Animals and other research organisms

Policy information about studies involving animals; ARRIVE guidelines recommended for reporting animal research, and Sex and Gender in Research

Laboratory animals	BALB/c nude mice (female, 12 weeks old), SD rats (male, 6 weeks old, weighed 180-220 g), Beagle (female, 2 years old, weighed 13.0 kg) were used as animal models in this work. The nude mice were kept at a temperature of 20 degrees Celsius, a humidity of 50, a diurnal temperature difference of no more than 3 degrees Celsius, and an alternating time of 12/12 between light and dark.
Wild animals	No wild animals were used in the study.
Reporting on sex	In order to avoid experimental differences caused by animal sex, animals of the same sex were used in the same experiment.
Field-collected samples	No field collected samples were used in the study.
Ethics oversight	The Institutional Animal Care and Use Committee of Zhejiang University and Zhejiang Academy of Medical Sciences

Note that full information on the approval of the study protocol must also be provided in the manuscript.
